# circFBXL5 promotes breast cancer progression by sponging miR‐660

**DOI:** 10.1111/jcmm.14737

**Published:** 2019-11-15

**Authors:** Huamao Zhou, Guohui Tang, Mi Zhao, Liming Xie, Yuanjie Xie, Zhiwei Zhang, Xiusheng He

**Affiliations:** ^1^ Key Laboratory of Cancer Cellular and Molecular Pathology in Hunan Province Cancer Research Institute University of Southern China Hengyang China; ^2^ Nanhua Affiliated Hospital University of South China Hengyang China

**Keywords:** breast cancer, circular RNAs, competitive endogenous RNAs, metastasis

## Abstract

Increasing studies have revealed that circular RNAs (circRNAs) play important roles in cancer progression. However, the potential involvement of circRNAs in breast cancer metastasis to lung is not clear so far. In this study, we conducted circular RNA microarrays of primary breast cancer tissues and lung metastatic tissues. The results revealed that circFBXL5 (hsa_circ_0125597) up‐regulated the most in lung metastatic tissues. Survival analysis revealed that high levels of circFBXL5 correlated with worse outcome of breast cancer. Further experiments showed that knockdown of circFBXL5 inhibited breast cancer cell proliferation and migration to lung. Mechanism study showed that circFBXL5 acted as a sponge for miR‐660 and compete binding to miR‐660 with SRSF6, leading to increased expression of SRSF6. Collectively, our study highlighted the regulatory function of the circFBXL5/miR‐660/SRSF6 pathway in breast cancer progression, which could be potential therapeutic targets for breast cancer.

## 1 INTRODUCTION

Recent studies have discovered abundant circular RNAs (circRNAs) in normal and malignant human cells and circRNA has become particularly hot field for cancer research.^1^ The regulatory transcriptional roles of circRNAs have been reported in multiple cancers. And circRNAs could be useful biomarkers for cancer diagnosis and therapy.^2^ However, the role circRNAs play in breast cancer is still not clear.

RNA transcripts, such as mRNAs, lncRNAs and circRNAs, are reported to serve as competitive endogenous RNAs (ceRNAs) in cancer regulation.^3^ Among them, circRNAs are highly stable and therefore have advantages as ceRNAs.^4^ And circRNAs are reported to play vital roles in cancer progression by functioning as miRNA sponges.^5^ In colon cancer, circRNA CCDC66 sponges suppressor miRNAs to induce cancer proliferation and metastasis.^6^ And circHIPK3 sponges miR‐124 to regulate cell growth.^7^ But the potential involvement of circRNAs in breast cancer metastasis to lung is not clear so far.

Here, we conducted circRNA microarrays of primary breast cancer tissues and lung metastatic tissues. We found circFBXL5 (hsa_circ_0125597) up‐regulated the most in lung metastatic tissues. Survival analysis revealed that high levels of circFBXL5 correlated with worse outcome of breast cancer. Further experiments showed that knockdown of circFBXL5 inhibited breast cancer cell proliferation and migration to lung. Mechanism study showed that circFBXL5 acted as a sponge for miR‐660 and compete binding to miR‐660 with SRSF6, leading to increased expression of SRSF6. The circFBXL5/miR‐660/SRSF6 pathway played vital role in breast cancer progression and could be potential therapeutic targets for breast cancer.

## 2 MATERIAL AND METHODS

### 2.1 Ethical standards

This study was approved by the Ethics Committees of Nanhua Affiliated Hospital and performed according to the Helsinki Declaration. All patients provided informed consents. Animal study was approved and performed according to the guidelines of Institutional Animal Care and Use Committee of Nanhua Affiliated Hospital.

### 2.2 Patients samples

Primary breast cancer tissues and lung metastatic tissues were collected from Nanhua Affiliated Hospital and subjected to circRNA microarray analysis. Breast cancer tissues of 150 patients were collected from Nanhua Affiliated Hospital and subjected to qRT‐PCR.

### 2.3 Microarray analysis

CircRNA microarrays were conducted with CapitalBio Technology Human CircRNA Array v2 and analysed with GeneSpring software V13.0 (Agilent). The result was log2 transformed and median centred by genes with CLUSTER 3.0 software and analysed with hierarchical clustering by average linkage.

### 2.4 Cell culture and transfection

Breast cell lines were purchased from American Type Culture Collection (ATCC). Cells were cultured according to the supplier's instructions. Cell authenticity was verified by DNA fingerprinting. siRNAs for circFBXL5, miR‐660 mimics and inhibitors were purchased from GeneCopoeia (Table S1).

### 2.5 Cell counting kit‐8 (CCK‐8) assay

Cells (1 × 10^3^) were seeded and 48 hours after transfection CCK‐8 solution (Dojindo Laboratories) was added. After incubation at 37°C for 2 hours, absorbance at 450 nM was measured.

### 2.6 Colony formation assay

Cells (1 × 10^3^ cells/well) were seeded and incubated for 2 weeks at 37°C. Colonies were fixed with methanol then stained with 0.1% crystal violet.

### 2.7 Mouse xenograft model

Cells (2 × 10^6^) were subcutaneously injected into the dorsal flanks of BALB/c nude mice (three mice per group, 4‐week‐old, female) and treated with an intratumoural injection (40 μL si‐NC or si‐circFBXL5) every 4 days. Xenograft tumours were excised 4 weeks later, and tumour weights were measured.

For lung metastasis, cells (1 × 10^5^) were injected through tail veins (three mice per group). The lungs were excised 8 weeks later, and the number of metastatic nodules was counted and validated by haematoxylin and eosin (HE) staining.

### 2.8 RNA immunoprecipitation (RIP) assay

Cells were transfected with MS2bs‐circFBXL5, MS2bs‐circBXL5‐mt or blank control using Lipofectamine 2000. RNA immunoprecipitation was conducted with a GFP antibody (Roche) and a Magna RIP RNA‐Binding Protein Immunoprecipitation Kit (Millipore) 48 hours later. And miR‐660 level was detected. RNA immunoprecipitation assay on Ago2 was performed with anti‐Ago2 antibody (Millipore) 48 hours after transfection, and the levels of circFBXL5, SRSF6 and miR‐660 were measured.

### 2.9 Statistical analysis

Statistical analysis was conducted using SPSS 19.0 software. Comparisons between groups were conducted using *t* tests. Survival analysis was conducted by Kaplan‐Meier plots and log‐rank tests. Data are presented as mean ± SD of three independent experiments, and *P* < .05 was considered statistically significant.

## 3 RESULTS

### 3.1 circFBXL5 is up‐regulated and related to worse outcome of breast cancer

**Figure 1:**
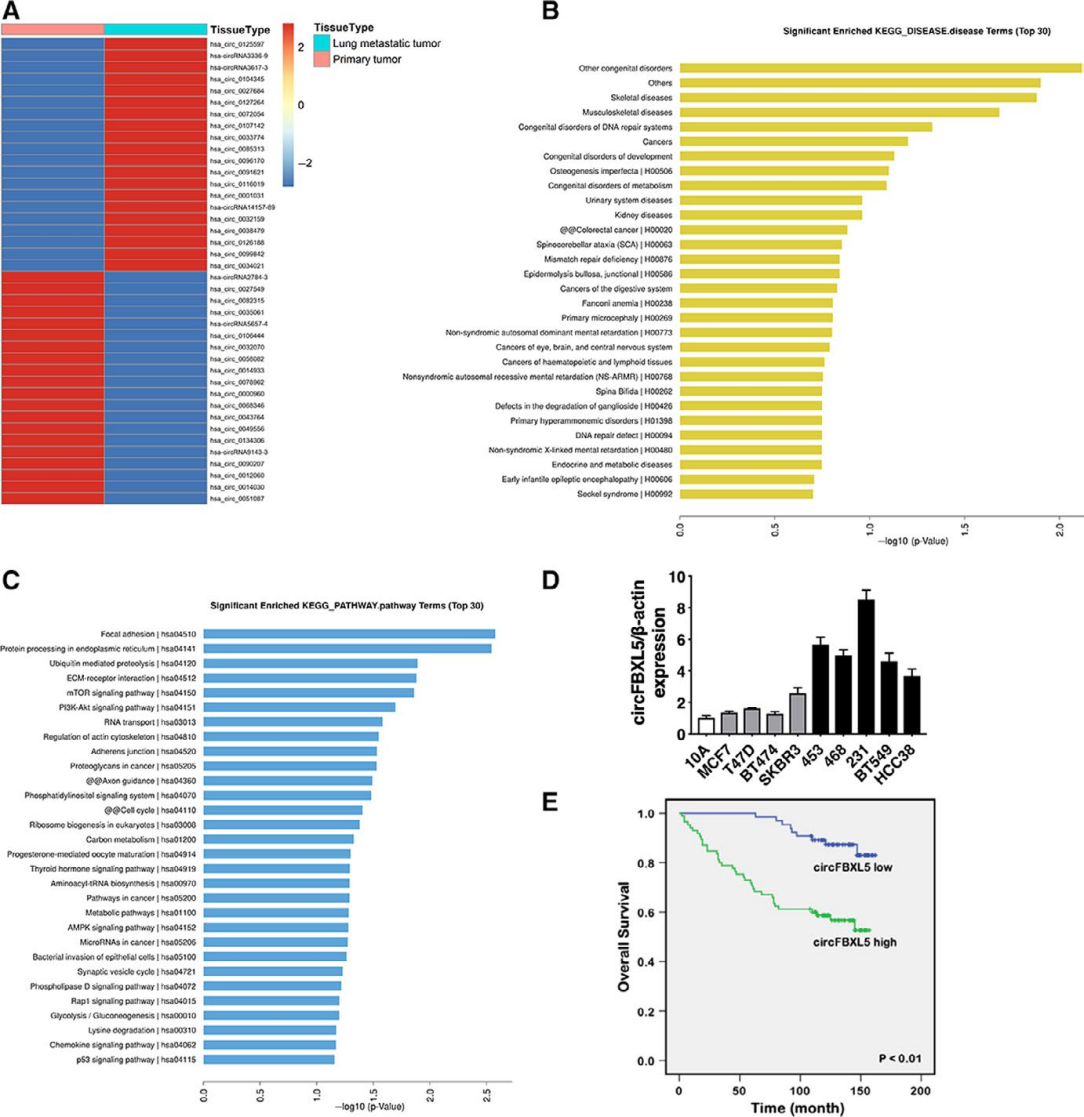
circFBXL5 is up‐regulated and correlated with poor outcome of breast cancer (A). Hierarchical cluster analysis showed the top 20 up‐regulated and down‐regulated circRNAs in lung metastatic tissues compared with primary breast cancer tissues: red, up‐regulated; blue, down‐regulated. B, KEGG disease analysis was performed. C, KEGG pathway analysis was performed. D, The expression of circFBXL5 in breast cancer cell lines. E, OS curves for 150 breast cancer patients with high or low circFBXL5 expression

To explore the potential involvement of circRNAs in breast cancer metastasis to lung, we conducted circRNA microarrays of primary breast cancer tissues and lung metastatic tissues. Figure 1A presented the top 20 up‐regulated and down‐regulated circRNAs based on fold change ≥2. Kyoto Encyclopedia of Genes and Genomes disease and pathway analysis of the linear mRNA transcripts corresponding to the circRNAs were conducted. The results revealed that the corresponding linear mRNAs were related to cancers (Figure 1B). Pathway analysis indicated cell adhesion and cell cycle, indicating the potential involvement in cell proliferation and migration progression (Figure 1C). Among the top 20 up‐regulated circRNAs, hsa_circ_0125597 up‐regulated the most in lung metastatic tissues and we therefore decided to study this circRNA. Hsa_circ_0125597 (chr4: 15632288‐15646331) was assumed to derive from F‐box and leucine rich repeat protein 5 (FBXL5) by human reference genome (GRCh37/hg19). Thus, we named hsa_circ_0125597 as ‘circFBXL5’.

We confirmed the expression of circFBXL5 and found that circFBXL5 was upregulated in breast cancer cell lines, especially in MDA‐MB‐453 and MDA‐MB‐231 (Figure 1D). Therefore, we used these two cell lines in the following study. To explore the clinical significance of circFBXL5 in breast cancer, we performed survival analysis on 150 breast cancer patients. circFBXL5 expression equalled to or greater than the average expression level was considered as ‘circFBXL5 high’ group. There were about 57% (85/150) of breast cancer patients had high circFBXL5 expression. Survival analysis revealed that high levels of circFBXL5 were related to worse outcome of breast cancer, indicating the vital role circFBXL5 plays in breast cancer progression (Figure 1E).

### 3.2 Knockdown of circFBXL5 inhibits breast cancer proliferation and migration

**Figure 2:**
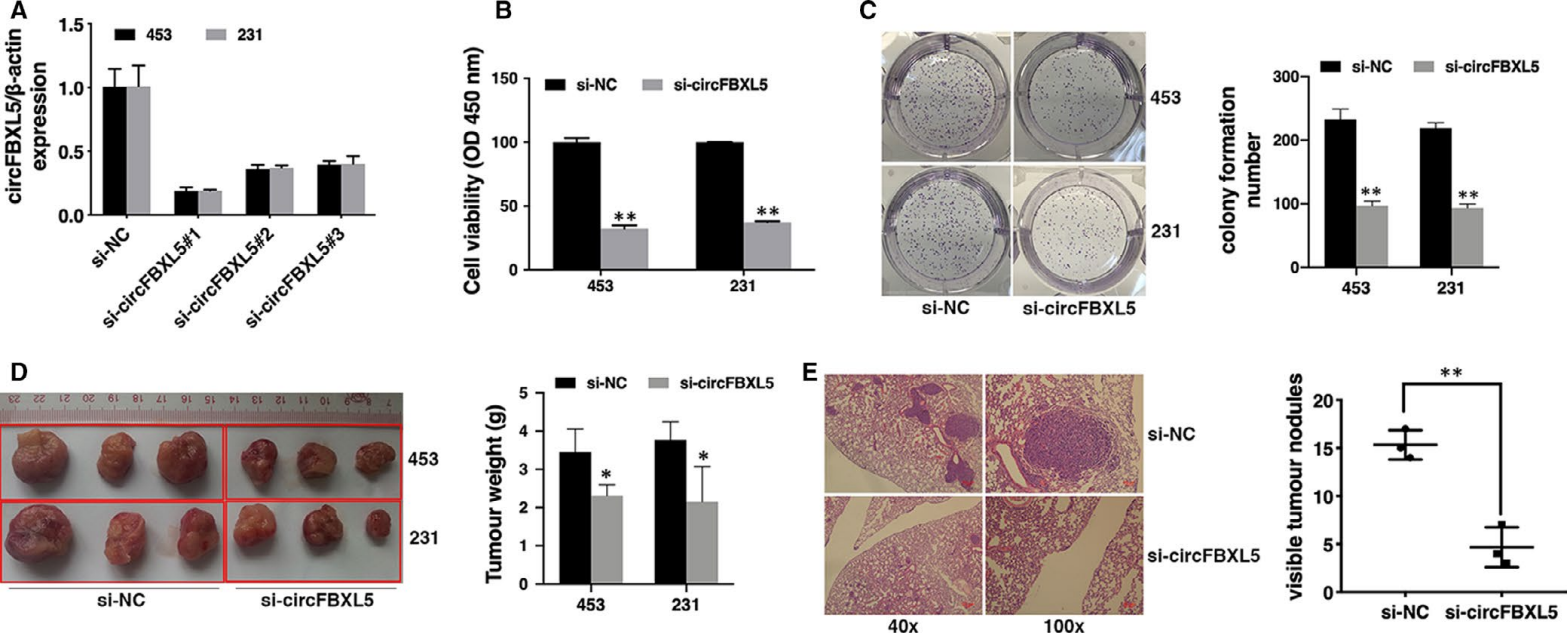
Knockdown of circFBXL5 suppresses proliferation and migration of breast cancer (A). si‐circFBXL5#1 successfully knocked down circFBXL5. B, CCK‐8 assay was performed to assess cell proliferation. C, Colony formation assay was performed to assess cell colony‐forming ability (left), and the colony formation number was quantified by ImageJ (right). D, Representative images of mouse xenografts tumours (left) and tumour weights were summarized (right). E, Representative images of lung metastatic nodules in HE‐stained sections (left). The number of metastatic nodules was quantified (right). *P < .05 and **P < .01

To investigate circFBXL5 functions in breast cancer, we knocked down circFBXL5 successful by si‐circFBXL5#1 (Figure 2A). CCK‐8 assay revealed that circFBXL5 down‐regulation suppressed cell proliferation (Figure 2B). And knockdown of circKIF4A suppressed breast cancer cell colony formation ability (Figure 2C).

To investigate circFBXL5 functions in vivo, we established mouse xenograft models. The results showed that circFBXL5 inhibition significantly decreased tumour growth (Figure 2D) and lung metastasis (Figure 2E), indicating that knockdown of circFBXL5 suppresses cell proliferation and migration in breast cancer.

### 3.3 circFBXL5 functions as a miR‐660 sponge

**Figure 3:**
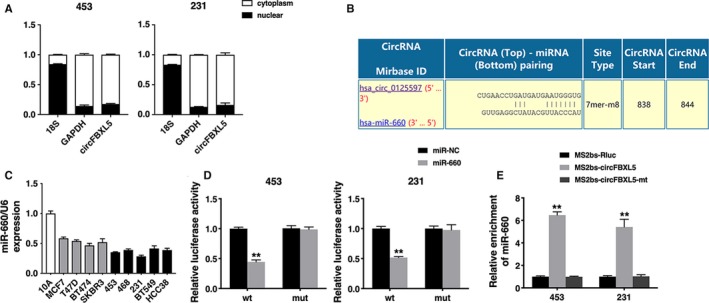
circFBXL5 acts as a sponge for miR‐660 (A). The levels of nuclear control transcript (18S), cytoplasmic control transcript (GAPDH) and circFBXL5 were assessed in nuclear and cytoplasmic fractions. B, The predicted binding sites of miR‐660 within the circFBXL5 sequence. C, The expression of miR‐660 in breast cancer cell lines. D, Luciferase assay of cells cotransfected with miR‐660 mimics and wild‐type or mutant luciferase reporter. E, MS2‐based RIP assay in cells transfected with MS2bs‐circFBXL5, MS2bs‐circFBXL5‐mt or control. **P < .01

Next, we explored circFBXL5 intracellular location and circFBXL5 was mainly localized in cytoplasm, indicating that circFBXL5 could act as a miRNA sponge (Figure 3A). Thus, circular RNA Interactome (https://circinteractome.nia.nih.gov/index.html) was used to predict the potential circRNA/miRNA interaction. We found binding sites of miR‐660 in circFBXL5 sequence (Figure 3B). And miR‐660 was down‐regulated in breast cancer cell lines (Figure 3C). Luciferase reporter assay showed that the luciferase activity decreased after transfected with wild‐type reporter and miR‐660 mimics (Figure 3D). To further confirm the binding between circFBXL5 and miR‐660, we conducted RIP assay. And miR‐660 was mainly enriched in RNAs retrieved from MS2bs‐circFBXL5, indicating that circFBXL5 might function as a miR‐660 sponge (Figure 3E).

### 3.4 circFBXL5 functions as a ceRNA for SRSF6

**Figure 4:**
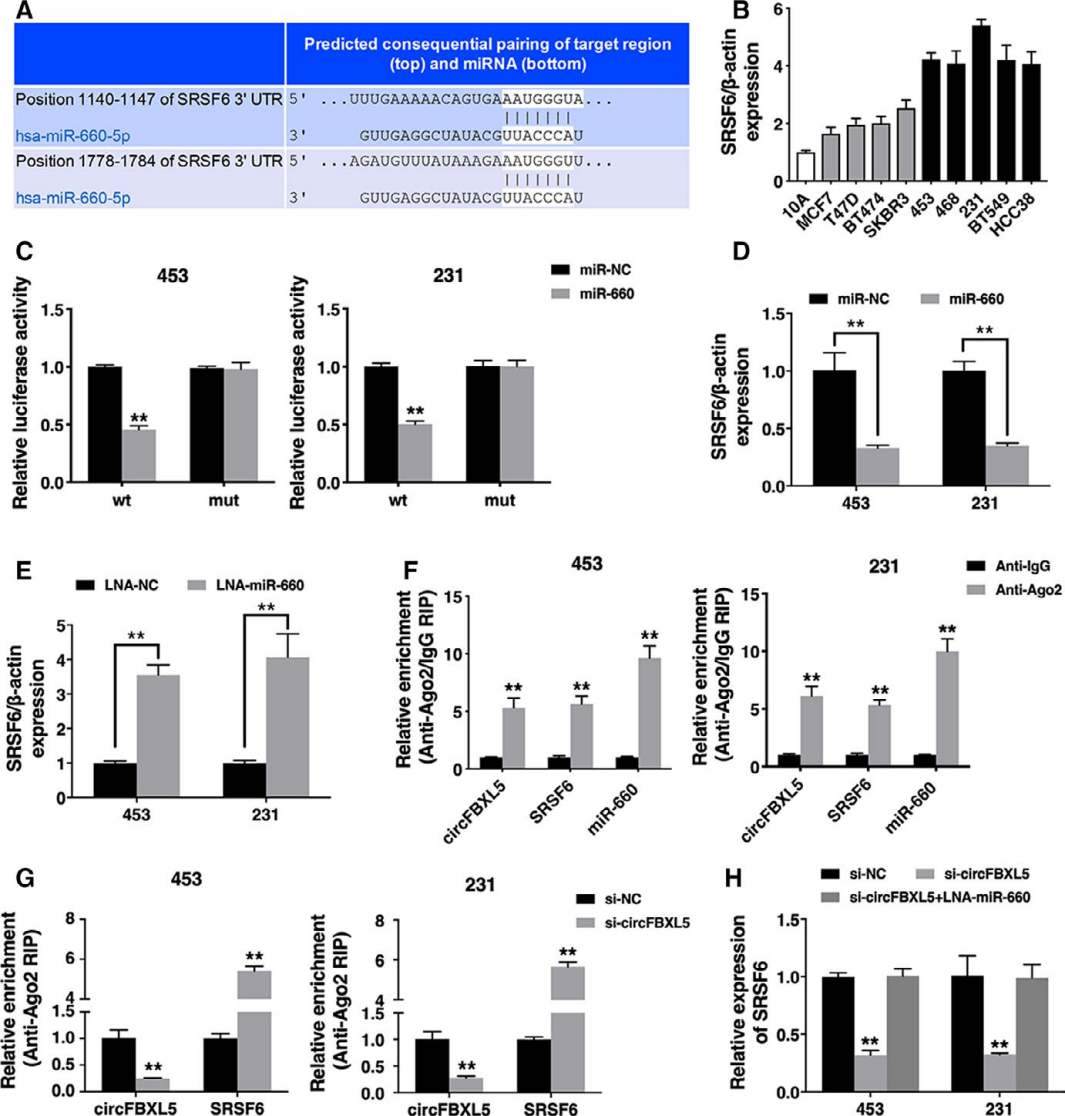
circFBXL5 acts as a ceRNA to regulate SRSF6 (A). The predicted binding sites of miR‐660 within the SRSF6 3′UTR. B, The expression of SRSF6 in breast cancer cell lines. C, Cells were transfected and luciferase assay was performed. D, Cells were transfected, and the expression of SRSF6 was detected. E, The expression of SRSF6 was detected. F, RIP assay showing the enrichment of circFBXL5, SRSF6 and miR‐660 on Ago2 relative to IgG. G, Cells were transfected and RIP assay on Ago2 was performed. H, Cells were transfected, and the expression of SRSF6 was detected. **P < .01

Next, we used TargetScan to find target genes of miR‐660, and serine and arginine rich splicing factor 6 (SRSF6) was predicted (Figure 4A). And SRSF6 was up‐regulated in breast cancer cell lines (Figure 4B). Luciferase reporter assay showed that the luciferase activity decreased after transfection with miR‐660 mimics and wild‐type reporter (Figure 4C). And the expression of SRSF6 was suppressed by miR‐660 and increased by miR‐660 inhibitor, indicating that SRSF6 is a target gene of miR‐660 and is regulated by miR‐660 (Figure 4D,E).

Moreover, RIP assay on Ago2 revealed that circFBXL5, SRSF6 and miR‐660 were all enriched to Ago2 (Figure 4F). Additionally, knockdown of circFBXL5 reduced circFBXL5 enrichment to Ago2, while increased SRSF6 enrichment to Ago2, which indicated that circFBXL5 acted as a SRSF6 ceRNA to compete binding with miRNAs (Figure 4G). Moreover, knockdown of circFBXL5 decreased the expression of SRSF6, but miR‐660 inhibitor could reverse this effect, indicating that circFBXL5 sponges miR‐660 to regulate SRSF6 expression (Figure 4H).

## 4 DISCUSSION

Increasing studies reveal that circRNAs are deregulated and play important roles in cancer progression.^8^ In breast cancer, circRNAs are also associated with clinical and biological properties. circCNOT2 was found associated with tumour proliferation, lymphocytic infiltration and patient outcome. And knockdown of circCNOT2 significantly reduced cancer cell viability.^9^ circEPSTI1 was significantly up‐regulated in triple‐negative breast cancer and related to worse outcome. circEPSTI1 knockdown suppressed cell proliferation and induced cell apoptosis.^10^ However, the potential involvement of circRNAs in breast cancer metastasis to lung is not clear so far.

In this study, we conducted circRNA microarrays of primary breast cancer tissues and lung metastatic tissues and found circFBXL5 (hsa_circ_0125597) up‐regulated the most in lung metastatic tissues. Survival analysis revealed that high levels of circFBXL5 were related to worse outcome of breast cancer. Further experiments showed that knockdown of circFBXL5 inhibited breast cancer cell proliferation and migration to lung, indicating that the vital role circFBXL5 plays in breast cancer progression.

It is reported that circRNAs could function as ceRNAs to sponge miRNAs and regulate cancer progression.^11^ In triple‐negative breast cancer, circAGFG1 acted as a ceRNA for miR‐195 to regulate cyclin E1 and promote cancer progression.^12^ And hsa_circ_001783 was reported to promote breast cancer progression via sponging miR‐200c.^13^ Here, we found that circFBXL5 had binding sites for miR‐660 and could function as a miR‐660 sponge.

As a tumour suppressor, miR‐660 is dysregulated in many cancers therefore may be a therapeutic approach for cancer. In renal cell carcinoma, miR‐660 was down‐regulated and could suppress cell migration, invasion and proliferation, and induce cell apoptosis.^14^ In gastric cancer, miR‐660 was significantly down‐regulated and closely related to poor outcome. And miR‐660 inhibited proliferation and induced apoptosis in gastric cancer.^15^ In lung cancer, miR‐660 was down‐regulated and correlated with poor prognosis. And miR‐660 reduced migration, invasion and proliferation and increased apoptosis.^16^ However, its role in breast cancer is currently unclear. Here, we found that miR‐660 was down‐regulated in breast cancer cell lines. Further experiment showed that SRSF6 was a target gene of miR‐660 and was regulated by miR‐660.

Recent study considers SRSF6 as an oncogene in tumour progression and is frequently overexpressed in cancers. In colorectal cancer, SRSF6 was up‐regulated and associated with poor prognosis. And SRSF6 could promote cell proliferation and metastasis.^17^ In lung and colon cancer, SRSF6 was overexpressed and could enhance cell proliferation and survival.^18^ However, the biological functions of SRSF6 in breast cancer are still unclear. Here, we found that SRSF6 was up‐regulated in breast cancer cell lines. And circFBXL5 could act as a ceRNA to compete binding with miR‐660, leading to increased expression of SRSF6.

Collectively, we found circFBXL5 up‐regulated and correlated with poor outcome of breast cancer. circFBXL5 sponged miR‐660 to regulate SRSF6 expression and breast cancer proliferation and migration. Our study highlighted the regulatory function of the circFBXL5/miR‐660/SRSF6 pathway in breast cancer progression, which could be potential therapeutic targets for breast cancer.

## CONFLICT OF INTEREST

The authors declare no conflict of interest.

## AUTHOR CONTRIBUTIONS

ZZ and XH designed the experiments. HZ and GT performed the experiments. MZ and LX analysed and interpreted the data. HZ and YX were the major contributors in writing the manuscript. All authors read and approved the final manuscript.

## Data Availability

The datasets used and analysed during the current study are available from the corresponding author on reasonable request.
